# Distributed Coding/Decoding Complexity in Video Sensor Networks

**DOI:** 10.3390/s120302693

**Published:** 2012-02-29

**Authors:** Paulo J. Cordeiro, Pedro Assunção

**Affiliations:** 1 Instituto Politécnico de Leiria/ESTG, Campus 2, Morro do Lena, Alto Vieiro, 2411-901 Leiria, Portugal; 2 Instituto de Telecomunicações, Delegação de Leiria, Campus 2, Morro do Lena, Alto Vieiro, 2411-901 Leiria, Portugal

**Keywords:** video sensors, real-time transcoding, complexity control

## Abstract

Video Sensor Networks (VSNs) are recent communication infrastructures used to capture and transmit dense visual information from an application context. In such large scale environments which include video coding, transmission and display/storage, there are several open problems to overcome in practical implementations. This paper addresses the most relevant challenges posed by VSNs, namely stringent bandwidth usage and processing time/power constraints. In particular, the paper proposes a novel VSN architecture where large sets of visual sensors with embedded processors are used for compression and transmission of coded streams to gateways, which in turn transrate the incoming streams and adapt them to the variable complexity requirements of both the sensor encoders and end-user decoder terminals. Such gateways provide real-time transcoding functionalities for bandwidth adaptation and coding/decoding complexity distribution by transferring the most complex video encoding/decoding tasks to the transcoding gateway at the expense of a limited increase in bit rate. Then, a method to reduce the decoding complexity, suitable for system-on-chip implementation, is proposed to operate at the transcoding gateway whenever decoders with constrained resources are targeted. The results show that the proposed method achieves good performance and its inclusion into the VSN infrastructure provides an additional level of complexity control functionality.

## 1. Introduction

Sensor networks are a recent field of research and fast technological development, combining a wide variety of sensor and networking technologies to capture all possible types of information from the physical environment, virtually without constraints in location and availability over time. In this context, wireless sensor networks (WSNs) are commonly comprised of a set of nodes, each one typically including a sensor, microcontroller, power supply and a wireless communication device [1]. There are many platforms and different deployments around the World, but sensor networks are still difficult to deploy and to keep within reliable operational conditions. The hardware/software requirements can be quite different, depending on the target applications and technology in use. For instance, an outdoor environmental sensor network may need to last operational and reliable for a very long time and rely on energy harvesting [2].

In the particular case of visual sensor networks (VSNs), the sensor nodes consist of a camera and a video encoder to compress the video data before transmission. VSNs are certainly among the most challenging sensor networking infrastructures to deploy, because the stringent requirements of low transmission bandwidth and low power consumption impose strong constraints on the visual sensors, particularly on video encoders [3].

There are many sensor networking applications that can significantly benefit from the presence of video information. These applications include either video-only sensor networks or sensor networking applications in which video-based sensors augment their traditional scalar sensor counterparts [4]. Examples of such applications include health-care monitoring, environmental monitoring, emergency response, robotics, surveillance applications, industrial process control, traffic avoidance and automated assistance for the elderly and Ambient Assisted Living, among others. Most of these applications use VSNs with different types of characteristics, which allow the use of quite diverse technological options [5].

In the past there was a great research focus on the sensors themselves, their characteristics and the optimization of different parameters, such as size, power consumption, processing power, memory, transmission rate, *etc.* [6]. However, there has been little concern with end-user devices and how the information gathered from a large amount of sensors can be delivered to such users. This is particularly relevant in the case of VSNs, where the amount of visual data collected by many sensors is huge (even compressed) and some type of common transmission link must be used at the VSN output connection to deliver all coded streams to remote user devices. If such devices are mobile terminals (e.g., remote real-time surveillance applications), then the coded video streams must also be constrained in order to obtain reduced computational complexity in decoding. Therefore, the challenge of such VSN framework is twofold: firstly, it is necessary to reduce the power consumption of the visual sensors, which can be achieved by reducing the computational complexity of the video encoding process without significant degradation of the coding efficiency; secondly, it is necessary to deliver low decoding complexity video streams to reduce power consumption of portable decoders and to extend the battery life.

This paper addresses the problems referred to above, *i.e.*, a review of efficient methods to control the complexity of video encoders in the light of their use in VSNs and a novel framework to gather video streams from multiple visual sensors with the capability of reducing their decoding complexity by using a novel type of video transcoding for this specific purpose.

The rest of the paper is organized as follows: Section 2 provides a short description of the main concepts of VSN video sensor nodes. Section 3 addresses the problem of video coding complexity for VSNs. Section 4 presents the proposed VSN architecture with distributed video coding/decoding complexity. Section 5 discusses the complexity associated with video decoding and Section 6 presents an algorithm for reduction of decoding complexity. Finally Section 7 presents the simulation results and Section 8 concludes the paper.

## 2. Video Sensor Networks—A Review

In the past, several different research topics have been addressed in the field of both generic WSNs [1,5,7] and more specific VSNs. Different open problems have been tackled, such as those related to resource requirements and management, lifetime of battery-operated camera nodes and energy consumption, on-board processing optimisation and data compression to cope with the available network bandwidth, among others. In the case of VSNs, these were initially devised as a set of small, inexpensive, battery operated nodes, interconnected, generally via wireless, with each other over a restricted transmission range. These VSN networks are different from traditional WSNs because the nodes are required to be equipped with very low power cameras. These camera-equipped nodes have the capability to capture visual information from the surrounding areas at variable rates, process the data on-board (e.g., compress) and transmit the captured data through the hop-by-hop communication infrastructure to a base-station (Sink) [4,8,9].

Typically, how to simultaneously provide video quality and energy efficiency from low resource nodes is the main problem, because VSNs generate a large amount of data, hence processing and transmission of video data causes them to consume a great quantity of energy. For instance, a single low-resolution image of QVGA (320 × 240) at 12 bits/pixel will generate 115,200 bytes of data which corresponds to a transmission rate of 23.04 Mbps, in case of video at 25 frames/sec. How to extend the lifetime of nodes and also to balance the energy consumption of the whole network, is a critical factor in VSNs. This imposes much tough stringent requirements on the network nodes and interconnection infrastructure than only the amount of available RAM memory on a typical wireless sensor node, as usually referred to as a relevant limiting factor in WSNs [10,11]. Therefore, it is widely accepted that the limited power supply in sensor nodes is the most relevant bottleneck in video communications using VSNs [10–13]. In the light of such limitations, the video compression algorithms used in visual sensor nodes must have low computational complexity and at the same time high compression efficiency in order to cope with limitations in the bandwidth available for communication. Furthermore it is worthwhile to note that the energy cost increases for higher communication rates. As an overall consequence of all these constraints, the quality of the visual information suffers from the limited power, processing capabilities, memory and available channel bandwidth in VSNs.

**Figure 1.**
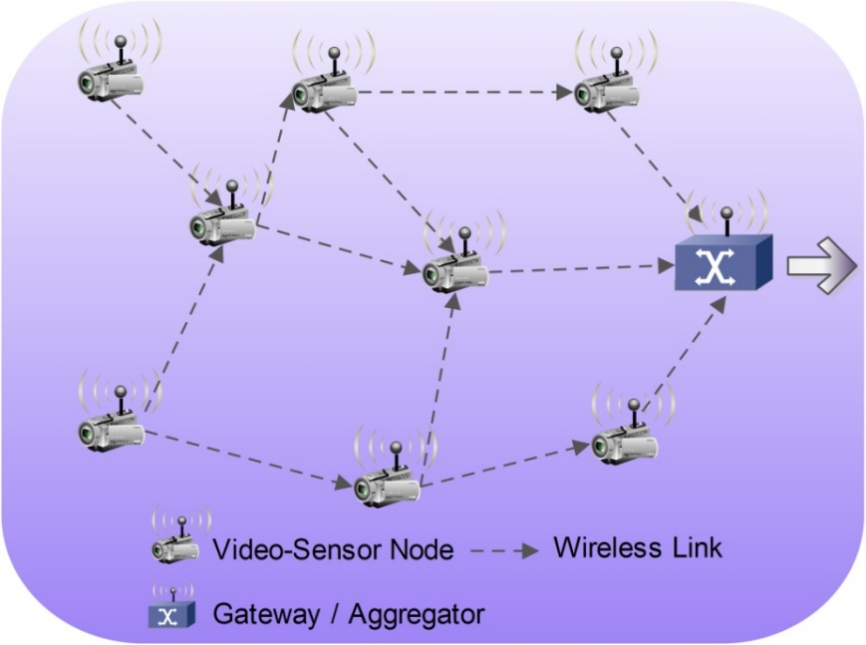
Typical self-organizing wireless VSN.

In the case of wireless VSNs, a specific dedicated communication infrastructure is not absolutely necessary, since these are self-organizing and highly dynamic structures, with each communication node serving as both server and router for data transmission [14–16]. A typical VSN is illustrated in Figure 1, where one can identify video sensor nodes acting as both interconnecting nodes and data acquisition/processing modules. In this case, a wireless network is assumed to be the interconnection infrastructure for communication between nodes.

### 2.1. VSN Architecture

**Figure 2.**
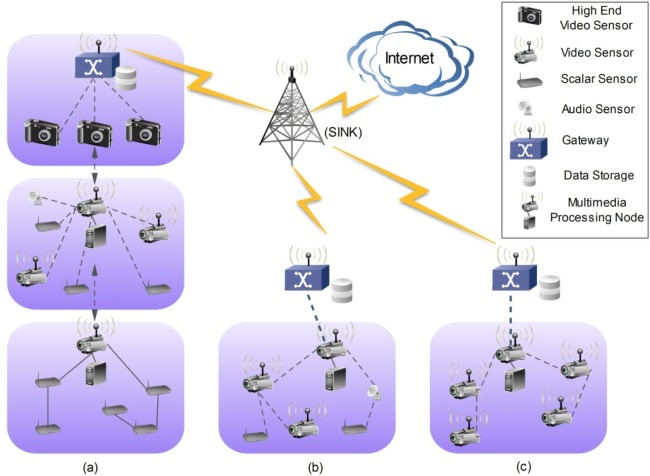
VSN Architecture.

The current VSN architectures are classified in three broad categories, according to the type of organization and clustering used for the sensors, which in turn depend on the envisaged application and also on the physical characteristics of the whole environment. A generic VSN is depicted in Figure 2 where the three main categories are defined as follows [5]:

- Single-tier flat architecture having homogeneous sensors.
- Single-tier clustered architecture having heterogeneous sensors.
- Multi-tier heterogeneous architecture with heterogeneous sensors support.

The multi-tiered architecture shown in Figure 2(a) is basically composed of several tiers with heterogeneous sensor nodes. In this Figure, a first tier with simple scalar sensors corresponds to the lower level of complexity, the second tier includes video sensors with medium resolution and the third tier has high-power sensor devices with enough resources to perform complex processing tasks. In this multi-tiered architecture, each tier may have a node with high processing and storage capability in order to deal with high complex processing tasks. Overall this architecture is more flexible than the others and also offers better scalability better coverage and better reliability [5].

The single-tier clustered shown in Figure 2(b) is composed of a set of heterogeneous sensor nodes which gather different types of information from the surrounding environment (*i.e.*, not only visual information, but also other types of physical variables). A central processing unit can be used in each cluster in order to deal with intensive processing tasks which would be too heavy for the sensor nodes with small processing/storage capacity. Then sensory information collected at the sensor nodes is also transmitted hop-by-hop to the storage/sink node via the gateway. This architecture can be used in a wide range of applications because the heterogeneous nature of the sensors is not restricted to any predefined type.

The single-tier flat architecture shown in Figure 2(c) is characterized by comprising a set of homogeneous sensor nodes using the same video sensors, processing modules and communication characteristics. The nodes can be used either for capturing different types of information or as processing modules. The information collected in the sensors is transmitted hop-by-hop to a centralized storage/sink node that is accessed via the gateway. The distributed processing capability of this architecture combined with its homogeneous nature acts in favor of a longer autonomous lifetime of the whole network, because of the easier management of the processing power and computational complexity allocated to each node.

### 2.2. Applications of VSN

A challenging application field of VSNs is remote surveillance, where the video content must be streamed to some central monitoring point. As mentioned before, this requires relatively complex processing (e.g., video compression) and high transmission bandwidth. In general, these networks are adapted to the environment dynamics and are able to respond timely to a user’s request. It is worthwhile to point out that such VSNs are not necessarily limited by the absence of infrastructure nor do they require central servers with large resources [3].

VSNs can also be used in environmental monitoring applications where sensors are deployed in remote and inaccessible areas over long periods of time, e.g., sandbank evolution or control of animal population. Often cameras are combined with other type of sensors in order to be triggered only when an event is detected by other sensors in the network [17–20]. Such scheme can extend the lifetime of the sensor devices, so that they remain available for long periods of time.

Elderly personal and health care monitoring is another field of application targeted by VSNs. In this case, the inclusion of additional telemedicine devices extend the use of VSNs to remotely monitoring patient’s health parameters such as blood pressure, body temperature, heart rate, breathing activity, and movements within a certain living area [21].

The VSN technology also enables applications where remote users can “visit” some location that is monitored by a set of video cameras–such schemes are usually known as telepresence systems [3]. Examples where such applications can be found include museums, galleries or exhibition rooms remotely accessed (e.g., via Internet) through live video streams generated by a set of visual sensors covering all interesting view points, and thus providing the sense of being physically present in those spaces [22].

Traffic congestion management is another potential growing application scenario for these networks. In general this type of application leads to large arrays of information sources as a result of the widespread use of information and communications technologies in road sensors, electronic toll collection devices, automatic video processing, global positioning systems, mobile sensor networks and smart phones. In this type of applications, VSNs play an important role to enable integration of diverse information about real time traffic and status for intelligent traffic routing. An example of a traffic monitoring system, using different types of sensory information with video playing a major role, is described in [23].

### 2.3. Video Sensor Nodes

The technology used in most video sensors and hardware platforms derives from other application fields, though particular attention is given to power consumption in the case of VSNs [5]. Nowadays, the increasing development of complementary metal-oxide semiconductor (CMOS) imaging sensors enables to capture and process optical images of quite different resolutions on a single chip. Compared to previous technology of charge-coupled device (CCD) image sensors, CMOS image sensors are less expensive and more energy efficient, which makes them better candidates for VSNs. The availability of CMOS image sensors drive the massive deployment of digital video cameras on resource constrained embedded wireless sensor nodes, leveraging the video sensing capabilities in wide scale deployments. Currently, most of available hardware platforms for video sensor nodes are mainly built to provide spatial resolutions from low range to medium resolution range.

**Figure 3.**
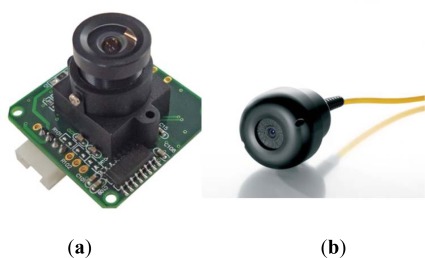
(**a**) Low-resolution (640 × 480) camera; (**b**) Medium resolution (800 × 600) camera.

The image quality provided by CMOS technology is now reaching the same level as CCD quality in the low and midrange, while CCD is still the technology of choice for high-end image sensors. The CMOS technology allows integrating a lens, an image sensor and image processing algorithms, including image stabilization and image compression, on the same chip. With respect to CCD, cameras are smaller, lighter, and consume less power. Hence, they constitute a suitable technology to realize imaging sensors to be interfaced with wireless nodes. Figure 3 shows hardware modules used for video acquisition in sensor networks.

## 3. Video Coding Complexity

As visual information deals with a considerable amount of data, video sensors must use compression to reduce the amount of data in order to effectively save storage memory and also to fit the data on the available network bandwidth. The H.264/Advanced Video Coding (AVC) is an industry standard for video compression (24 January 2004; v3 (with FRExt), September 2004; v4, July 2005 #599). The H.264/AVC standard, also known as MPEG-4 Part 10, is a successor to earlier standards such as MPEG-2 and MPEG-4 Visual. The H.264/AVC achieved the target to double the coding efficiency (halving the bit rate necessary for a given level of fidelity) in comparison to any other existing video coding standards for a broad variety of applications. This coding efficiency improvement comes at a cost of significant increased codec complexity, which increases the hardware requirements and power consumption for encoding and decoding.

As in previous video coding standards, the H.264/AVC video codec defines the video signal structure as a series of groups of pictures (GOPs) comprising frames of luminance (Y) and two chrominance (Cb and Cr) pixels. Each individual picture is organized into slices which in turn are divided into non-overlapping blocks, *i.e.*, macroblocks (MBs). Slices are typically encoded as either Intra (I), Predictive (P), or Bi-Predictive (B) where I slices are encoded using predictive pixels from the same frame and are independent of all others, P slices use inter-frame prediction methods as well as intra-prediction methods and B slices use an expanded set of forward and/or backward inter-prediction methods compared to P-frames. For P and B slices, motion estimation (ME) is carried out for each MB to find the best match from a reference frame. In H.264, each MB can be further partitioned into sub-blocks and multiple reference frames can also be used. An integer transform (IT), with similar characteristics to those of the discrete cosine transform (DCT) used in previous standards, is applied to the block residue, concentrating most of the block energy into the low frequency region. Quantization and entropy coding of the remaining coefficients are then applied to further reduce irrelevancy and redundancy of the video signal.

In H.264/AVC most of the coding efficiency comes from exploiting temporal redundancies within the video sequence by performing block based motion compensated prediction. However, motion estimation is only performed at the encoder, and the motion vectors have to be explicitly coded into the bit stream. Motion estimation is among the most complex operations in H.264/AVC video coding because of the huge number of operations involved in finding the best match for each macroblock (MB) or block. Also on the decoder side, previous studies on H.264/AVC decoder complexity have shown that motion compensation (MC) is the most computationally complex functional block at the decoder, followed by the deblocking filter process [25,26], as will be quantified in Section 6.1, thus this is also the most energy consuming task.

### 3.1. Video Coding for VSN

Video coding for the VSN must be done in a different manner from the coding for video telephony or other multimedia applications. This difference is mainly due to two features associated to the sensor networks: (1) limited resources and (2) data quality. The first feature is a critical aspect in VSN since video cameras collect a huge amount of data that must be transmitted over a wireless link, as pointed out before. Therefore video compression must be used to reduce the amount of data to be transmitted, but this can only be obtained at very high computational cost. The second feature leads to the fact that while video compression algorithms often have very powerful data reduction capabilities, they introduce significant distortion [27]. When the network gateway decodes the video stream and analyses the data, the distortion introduced by compression can heavily bias the results, thus possibly leading to wrong interpretations. Therefore, video coding for sensor networks requires novel perspectives to cope with such issues [28].

A different paradigm that fits the requirements of VSNs is Distributed Video Coding (DVC) [29,30]. The DVC fundamentals are inherited from the Distributed Source Coding (DSC) [31] fundamentals which refer to separate encoding at a number of sensor nodes and joint decoding at the base station (or gateway). DVC is based on information theory results demonstrated in the seventies, by the Slepian-Wolf [32] and the Wyner-Ziv theorems [33]. Under this paradigm, every encoder should operate with low power consumption and independent of other sensor nodes, while the decoder has enough resources to exploit the correlation existing between the different encoded bitstreams. Slepian-Wolf and Wyner-Ziv theorems show that DSC can achieve the same or similar rate-distortion performance to traditional (non-distributed) source coding. A DVC solution developed in the past, known as PRISM (Power-efficient, Robust, hIgh compression, Syndrome-based Multimedia coding) [30,34], includes motion estimation at the decoder, which eases the coding complexity at the encoding node. The studies conducted with PRISM have shown that better video quality than H.264/AVC can be achieved in lossy wireless links. Therefore the use of DVC to make use of the combined processing power of neighboring sensors with some degree of overlapping in the visual information appears to be and effective approach to be used in energy-constrained video coding applications. Moving the motion estimation function from the encoder to the decoder has provided good results in reducing encoding complexity while keeping coding efficiency [31,35].

## 4. Proposed VSN with Distributed Video Coding/Decoding Complexity

**Figure 4.**
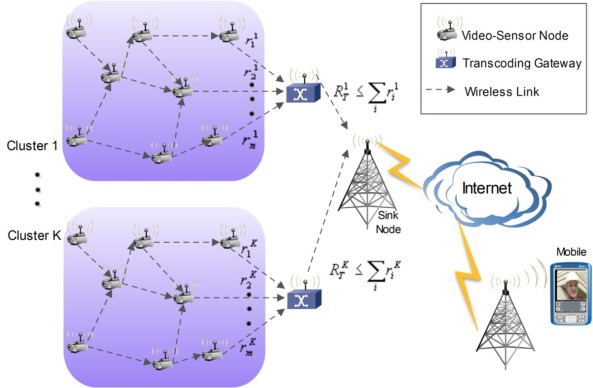
Proposed VSN architecture.

The proposed VSN architecture with distributed complexity control capability is depicted in Figure 4. In such an architecture, there are several clusters of video sensors, interconnected through a mesh network which has some outgoing links to a centralized transcoding gateway. Among other important functionalities, the gateway may perform stream multiplexing and transmission to a core network or Internet, which in turn is used as the core network to deliver the video data to the target receivers. Among many possible receivers that can be used, mobile or portable devices are among those with more stringent limitations in regard to processing power and energy constraints. Therefore, the network architecture takes into account the possibility of having end-user devices with constrained computational complexity in order to achieve extended battery life. This is the case, for instance, of video surveillance applications where remote users can have portable/mobile devices for real-time monitoring of the area of interest. Each cluster of video sensors can be associated with a certain specific application or geographical area, so the architecture is flexible and scalable to fit a wide variety of requirements. As pointed out before, the complexity constraints are mainly located at both extremes of the whole framework, *i.e.*, the video sensors and the end-user terminals. The transcoding gateway is responsible for matching the complexity requirements of both ends.

### 4.1. Transcoding Gateway

**Figure 5.**
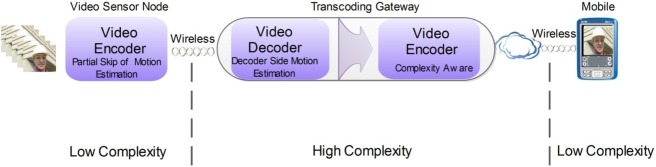
Transcoding gateway.

The general architecture of the transcoding gateway is shown in Figure 5. It is comprised of a cascade of decoder-encoder where each one has specific functionalities that enable management of the computational complexity allocated to the video sensor and mobile decoder, to a certain extent. In the case of the video sensor, the encoding algorithm may skip the motion estimation function (either totally or partially) in order to reduce the computational complexity required at the sensor device. As a consequence the transmitted bit rate is also lower than in the case where motion vectors are sent in the coded stream. Then the decoder of the transcoding gateway must implement the same motion estimation function as the encoder of the video sensor, in order to build the same predictions as those used for encoding. Recent work has shown that motion estimation can be efficiently performed at the decoder [36,37]. Either decoder-side motion vector derivation or decoder-side motion estimation can lead to significant reduction in coding rate and at the same time transferring part of the computational complexity from the encoder to the decoder. Therefore, this transcoding process is equivalent to transferring a significant part of the encoding complexity from the video sensor to the transcoding gateway, which is beneficial to the sensor and to the transmission efficiency without any problem to the gateway, because this is wired powered equipment without major computational or energy constraints. It is worthwhile to notice that such a video coding scheme is not compliant with any currently available standard, but this is not a problem because the communication between the VSN nodes and the transcoding gateway do not need to be standard compliant. Compatibility is only necessary between transcoding and the video sensors, which means that manufacturers of video sensors must also provide a compatible transcoder, which can be built as a standalone system-on-chip and integrated in different types of equipment.

The video encoder of the transcoding gateway plays the important role of matching the output rate to the bandwidth constraints of the outgoing transmission channel, *i.e.*, transrating the input stream, and also takes into account the type of end-user terminal. In the case of mobile or portable devices, the encoder activates its complexity-aware coding mode in order to produce streams with low decoding complexity. As a consequence the bit rate might be slightly increased, while the decoding complexity can be significantly reduced. In the next sections a decoding complexity-aware method for standard video encoders is proposed and described in detail, along with results and discussion.

## 5. Video Decoding Complexity

Current portable devices such as mobile phones, personal digital assistants, palmtops, *etc*. are increasingly used to access, decode and render multimedia content in which compressed video plays a major role. The limitation imposed by battery life and computational constraints of processing equipment have been a strong motivation for research on complexity issues of video coding and decoding systems in the last few years [38–40]. Extending the time that multimedia content can be delivered and consumed in portable equipment is a desirable feature for both users and VSN-based applications which may be achieved through different approaches. Reduction of the computational complexity [41] and power saving mechanisms [40] are among the most popular approaches to deal with this issue. Although power savings in portable devices is dependent on the characteristics of several different functional components of the device itself such as hardware, operating system, processing software implementation, communication protocols, *etc*., in video decoders this is highly related to the computational complexity of the decoding process. It is particularly relevant in the case of H.264/AVC video coding standard where complexity, at both the encoding and decoding sides, is a major concern in any practical real time implementation. Besides all implementation optimizations that can be considered for the purpose of minimizing decoding complexity, there is still room for further reduction by producing compressed video streams such that their inherent decoding complexity is made lower [42,43]. This can be done by constraining the encoder in order to reduce the use of the most complex coding tools and options which lead to higher decoding computational complexity. The challenge is how to achieve this goal without compromising too much the signal quality when compared with unconstrained encoding.

The decoding complexity of H.264/AVC streams is mainly because of motion compensation due to sub-pixel accuracy computations which demand for a great deal of processing. This is because computation of predictions from sub-pixel motion vectors is done by interpolation filters which need a different amount of filtering operations according to the location of the sub-pixel to be determined. In the next section we propose a measure of decoding computational complexity based on the amount of filtering operations needed to compute sub-pixels in order to account not only for rate and distortion but also for decoding complexity in the process of selecting the best motion vector for each block. Then the rate-distortion complexity performance is evaluated and compared with the normal rate-distortion optimized coding.

## 6. Algorithm for Decoding Complexity Reduction

Video encoding complexity has been a research issue in the last decades, either when new standards or coding algorithms emerge with more efficient coding tools than their main predecessors. Since on the decoder side, the problem of complexity is strongly dependent on the encoding side, different approaches can be used to limit the computational complexity required to decode video streams. For instance, in the MPEG-4 visual standard a video buffer verifier mechanism was defined to bound the decoding complexity of coded streams [44]. Another approach is based on producing compressed video streams with intrinsic low decoding complexity requirements [41]. This is implemented by modeling the most complex decoding functions and then by using such complexity models in the encoding constraints in order to produce light-decoding video streams [42,45].

### 6.1. Motion Compensation in H.264

Motion estimation is among the most complex operations in H.264/AVC video coding because of the number of operations involved in finding the best match for each MB or block. Also on the decoder side, previous studies on H.264 decoder complexity have shown that motion compensation (MC) is the most computationally complex functional block at the decoder, followed by the deblocking filter process.

**Figure 6.**
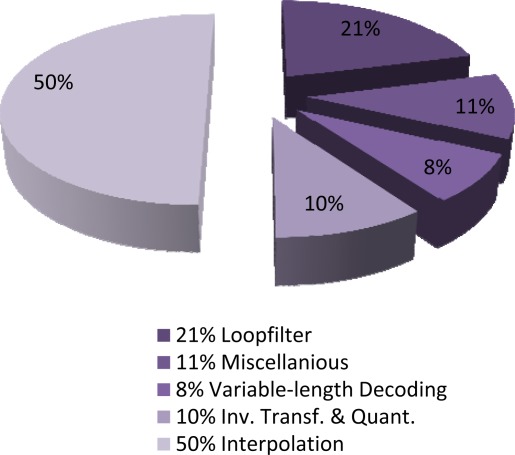
Computational complexity distribution in a typical H.264/AVC decoding process.

High complexity in motion compensation is mainly due to the interpolation needed to decode motion vectors with half or quarter pixel accuracy. For the baseline H.264 decoder, it was shown that interpolation takes around 39% of the execution time on average, and it can go up to 44% for some sequences. Figure 6 shows the breakdown of the complexity of a typical H.264/AVC decoder implementation as reported in [25].

### 6.2. Decoding Complexity Cost

In H.264/AVC video coding, the bit allocation and rate control mechanisms dynamically adjust the encoding parameters to achieve a target bit budget at the highest possible video quality. This is done through rate-distortion Lagrangian optimization in two coding stages: motion estimation and mode decision. In the first one, motion estimation, for each block B with a block mode M, the motion vector associated with the block is selected through a rate-distortion joint cost function:

(1) $$J_{\mathrm{Motion}}^{R,D}=D_{\mathrm{DFD}}+\lambda_{\mathrm{Motion}}R_{\mathrm{Motion}}$$

Equation (1), D_DFD_ is the prediction error, computed as either the sum of absolute differences (SAD) or the sum of squared difference (SSD), R_Motion_ is the estimated bit rate to encode the corresponding motion vector, λ_Motion_ is the Lagrange multiplier to control the weight of the bit rate cost relative to the prediction error. $J_{\mathrm{Motion}}^{R,D}$ is the rate-distortion joint cost comprising both R_Motion_ and D_DFD_. Since in general the search space for motion vectors is very large and SAD has lighter computation cost than SSD, the former is used more often.

In this work, in order to favour motion vectors with less interpolation complexity and penalize the ones with higher complexity, the conventional cost function presented in Equation (1) is added with a specific Lagrange term to model complexity cost. This allows the motion vectors to be selected based on a joint cost function which takes the three parameters into account, *i.e.*, a rate-distortion-complexity joint cost function as given by Equation (2):

(2) $$J_{\mathrm{Motion}}^{R,D,C}=J_{\mathrm{Motion}}^{R,D}+\lambda_{\mathrm{CMotion}}\;C_{\mathrm{Motion}}$$

where C_Motion_ is the complexity cost associated with the selected motion vector and λ_CMotion_ is the Lagrange multiplier for the complexity term. $J_{\text{Motion}}^{R,D}$ is the rate-distortion defined in Equation (1) and $J_{\text{Motion}}^{R,D,C}$ is the rate-distortion-complexity cost function. A similar approach was followed in [42].

The MB mode is directly related to the computational complexity because it defines which motion vector should be associated with each of its sub-blocks. For each motion vector, a prediction block must be computed by the decoder in order to reconstruct a predicted MB from all sub-blocks. After all inter mode candidates have an associated motion vector, the coding results of the modes are compared and the one that minimizes the following Lagrangian cost function is chosen according to Equation (3):

(3) $$J_{\mathrm{Mode}}^{R,D,C}=J_{\mathrm{Mode}}^{R,D}+\lambda_{\mathrm{CMode}}\;C_{\mathrm{Mode}}$$

where *C_Mode_* is the complexity cost associated with the selected block mode and *λ_CMode_* is the Lagrange multiplier for the complexity term. $J_{\mathrm{Mode}}^{R,D}$ is the rate-distortion as it was defined in Equation (1), with Motion replaced by Mode, and $J_{\mathrm{Mode}}^{R,D,C}$ is the rate-distortion-complexity cost function. Considering two extreme cases of *λ_CMotion_* = *λ_CMode_* = 0 the solution for Equations (2) and (3) is identical to that of Equation (1), *i.e.*, the complexity cost is not considered and motion vectors are chosen based only on the rate-distortion cost without taking into account the inherent decoding complexity of half and quarter-pixel accuracy motion. At the other extreme, *λ_CMotion_* = *λ_CMode_* = 1 results in minimum decoding complexity because complexity cost is dominant. However in this case the rate-distortion performance drops, thus the best solution is to tradeoff between rate, distortion and complexity using 0 < *λ_CMotion_* = *λ_CMode_* < 1, *i.e.*, varying the Lagrange multiplier between 0 and 1 for the complexity term, in both Equations (2) and (3).

### 6.2. Experimental Results

**Figure 7.**
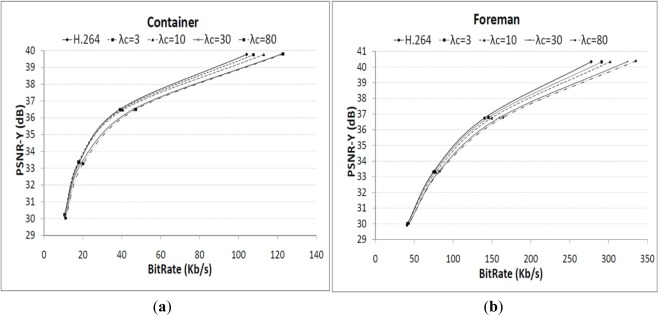
Rate-PSNR performance for different values of ***λ_C_***. (**a**) Container; (**b**) Foreman.

The efficiency of the proposed constrained coding method was experimentally evaluated in order to assess how decoding complexity can be reduced and how much quality drop is observed. Two sequences (Container and Foreman) with different types of motion were used in order to assess the influence of the video signal characteristics on the performance of the proposed method. The baseline mode of H.264/AVC was used as this is more appropriate to mobile devices. The experimental setup was defined according to the recommended simulation conditions for coding efficiency experiments [46,47]. As shown in Figure 7, the proposed method produces a small drop in PSNR for a wide range of bit rates, in comparison with normal H.264/AVC rate-distortion optimization.

The difference is greater for higher values of *λ_C_* because the choice for integer motion vectors increase while sub-pixel interpolation decreases because the complexity weight is higher in the Lagrangian cost function. Table 1 shows the processing time as a measure of the decoding computational complexity, for the same values of *λ_C_* as used in Figure 7. The rate-distortion optimization of the H.264/AVC is used for reference (2nd column) and the percentage of complexity reduction shown as *λ_C_*. As it can be seen in this table, the small decrease in rate-distortion (*i.e.*, less than 0.5 dB) performance shown in Figures 7(a,b) corresponds to significant savings in decoding computational complexity. Therefore the proposed method can be used for coding video streams with low computational complexity which is particularly useful for reducing the power consumption in portable devices.

## 8. Conclusions

In this paper we have proposed a novel architecture for VSNs where the computational complexity is taken into consideration. A new networking element, the transcoding gateway, was presented as a central processing, which can concentrate part of the encoding complexity from the video sensors and reduce the decoding complexity required from the end user terminal. Such a framework has high flexibility and scalability, making it suitable for many different applications. Furthermore we have also proposed an algorithm to produce low decoding complexity streams at the transcoding gateway. The results show that a significant complexity reduction can be achieved at the expense of a small reduction in coding efficiency. This algorithm is currently under implementation as a standalone processing module using FPGAs.

## Figures and Tables

**Table 1.** Average decoding complexity.

**Sequence**	**RD (ms)**	**R-D-C (*λ_C_* = 3)**		**R-D-C (*λ_C_* = 10)**		**R-D-C (*λ_C_* = 30)**		**R-D-C (*λ_C_* = 80)**	
		**ms**	**ΔC**	**ms**	**ΔC**	**ms**	**ΔC**	**ms**	**ΔC**
Foreman	1174.6	1,098.3	6.5%	1057.3	10.0%	974.3	17.1%	867.6	26.1%
Container	520.6	470.6	9.6%	457.6	12.1%	428	17.8%	440.3	15.4%
